# The relationship of left ventricular function to infarct surface area and volume

**DOI:** 10.1186/1532-429X-11-S1-P52

**Published:** 2009-01-28

**Authors:** Al-Hakim Ramsey, Michael C Fishbein, Thomas O'Donnell, James Sayre, Kalyanam Shivkumar, Alan Kadish, Paul Finn, Carissa G Fonseca

**Affiliations:** 1 University of California Los Angeles, Los Angeles, CA USA; 2 Siemens Corporate Research, Princeton, NJ USA; 3 Northwestern University, Chicago, IL USA

**Keywords:** Left Ventricular Function, Left Ventricular Mass, Volume Shrinkage, Impaired Left Ventricular Function, Free Open Source

## Objective

To examine the relationship between left ventricular (LV) myocardial function and myocardial infarct (MI) surface area and volume.

## Background

Delayed-enhancement MRI (DE-MRI) has been used successfully to describe the relationship between the volume of scar tissue and LV function [1]. Due to volume shrinkage of hyperenhanced tissue over time, DE-MRI derived infarct surface area (iSA) and the iSA to infarct volume ratio (iSA/V) may provide more stable indices of the extent of infarction than does scar volume and may better predict LV functional impairment.

## Methods

**Figure 1:**
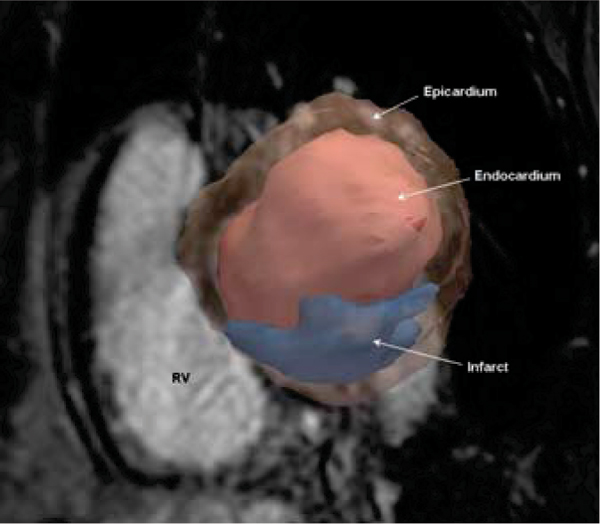
**3D rendering of LV and infarct (infarct mass: 14.1 g, iSA: 52.9 cm**^**2**^**, iSA/V: 3.9 cm**^**2**^**/ml) superimposed on a basal LV short-axis DE-MR image**.

Segmented SSFP cardiac cine and multi-shot inversion recovery spoiled gradient echo DE-MR images were obtained at 1.5 T, in 13 patients (11 M, 2 F; aged 40–82 years, mean 60.3 ± 12.3 years) with documented myocardial infarction from the Defibrillators To Reduce Risk by Magnetic Resonance Imaging Evaluation (DETERMINE) study. Cine and DE images were acquired in corresponding short-axis planes (spatial resolution ≤ 2.5 × 2.0 × 10.0 mm; slice gap ≤ 2 mm) from the base of the heart to the apex. 3DSlicer v2.6, free open source image analysis software (slicer.org), was used to manually segment and create a three-dimensional model of the infarct from which volume and iSA were derived (Fig 1).

LV function was assessed using CIM software [2], which provided the following global measurements: LV mass, LVEF, LV end-diastolic volume (EDV), and LV end-systolic volume (ESV). CIM also provided segmental data based on a 16 segment LV model: myocardial thickening (peak thickness – minimum thickness), EDV, ESV, and EF. Infarct and LV mass were calculated from their respective volumes assuming a constant density of 1.04 g/ml. Mid-ventricular segments that visually contained greater than 50% hyper-enhancement on DE-MRI were considered infarcted. Segments which were non-infarcted and not adjacent to infarcted segments were considered remote. Statistical comparisons between infarct and remote regions were made using a paired, two-tailed student's t-test. For linear relationship comparisons, the standard error of the estimate was calculated to examine goodness-of-fit.

## Results

**Figure 2:**
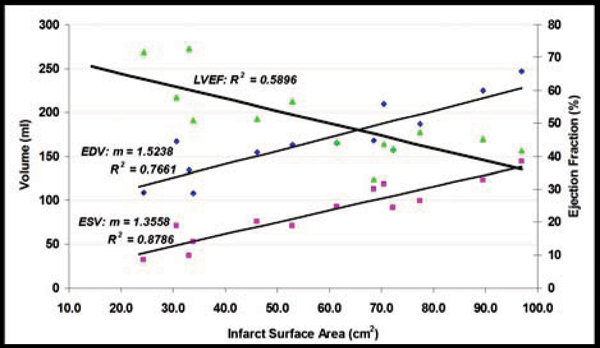
**Relationshop of LV volumetric measurements to infarct surface area**.

Infarct mass correlated with iSA (r = 0.958). EDV and ESV both increased with iSA (r = 0.875 and 0.937 respectively; Fig 2). ESV correlated more strongly with iSA than with relative infarct mass (100*infarct mass/LV mass; r = 0.499; p < 0.05). LVEF decreased with relative infarct mass (r = -0.526) and showed a higher correlation with iSA (r = -0.768, p < 0.01). Relative infarct mass correlated with iSA/V (r = -0.845). A relationship was not observed between iSA/V and LVEF (r = 0.298).

**Figure 3:**
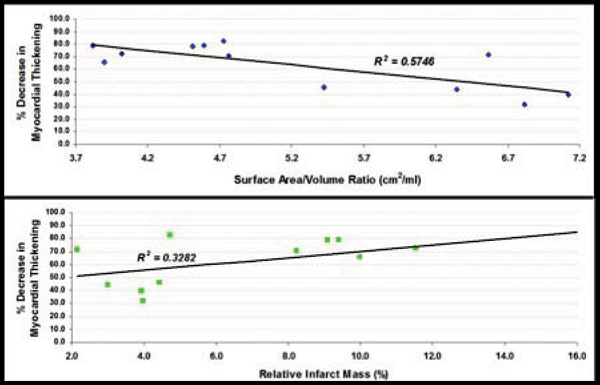
**Decrease in LV wall thickening in relation to infarct surface area to volume (iSA/V) ratio and relative infarct mass**.

Thickening of the infarcted region was reduced compared with the remote region (3.47 mm ± 1.95 vs. 9.79 mm ± 2.47, p < 0.001). The percent reduction in thickening of infarcted myocardium showed a negative correlation with iSA/V (r = -0.758) and a positive correlation with relative infarct mass (r = 0.573; Fig 3), the correlation with iSA/V being stronger (P < 0.0001).

## Conclusion

Impaired LV function is strongly related to infarct surface area. Relative infarct mass may underestimate the extent of damage post-MI because of thinning of infarcted myocardium during remodeling. Infarct surface area is less affected by volume shrinkage, and may be more stable over time than infarct mass in measuring the loss of functioning myocardium.

A greater decrease in LV wall thickening is associated with a larger relative infarct mass and surface area but a smaller iSA/V. A transmural increase in infarct size may increase infarct volume with little change in iSA. Infarct transmurality greater than 50% is associated with severe loss of myocardial function and mortality [3]. Thus, iSA/V can provide an objective quantitative index of transmurality, superior to relative infarct mass for predicting decrease in myocardial function.
